# Validation of Inter-Reader Agreement/Consistency for Quantification of Ellipsoid Zone Integrity and Sub-RPE Compartmental Features Across Retinal Diseases

**DOI:** 10.3390/diagnostics14212395

**Published:** 2024-10-27

**Authors:** Jordan Bell, Jon Whitney, Hasan Cetin, Thuy Le, Nicole Cardwell, Sunil K. Srivasatava, Justis P. Ehlers

**Affiliations:** 1 Cleveland Clinic Lerner College of Medicine Program, Case Western Reserve University, Cleveland, OH 44106, USA; 2 The Tony and Leona Campane Center for Excellence in Image-Guided Surgery and Advanced Imaging Research, Cleveland Clinic, Cleveland, OH 44195, USA; 3 Vitreoretinal Service, Cole Eye Institute, Cleveland, OH 44195, USA

**Keywords:** optical coherence tomography, age-related macular degeneration, diabetic macular edema, retinal disease, ellipsoid zone, retinal pigment epithelium, Bruch’s membrane, retina

## Abstract

Background: An unmet need exists when clinically assessing retinal and layer-based features of retinal diseases. Therefore, quantification of retinal-layer-thicknesses/fluid volumes using deep-learning-augmented platforms to reproduce human-obtained clinical measurements is needed. Methods: In this analysis, 210 spectral-domain optical coherence tomography (SD-OCT) scans (30 without pathology, 60 dry age-related macular degeneration [AMD], 60 wet AMD, and 60 diabetic macular edema [total 23,625 B-scans]) were included. A fully automated segmentation platform segmented four retinal layers for compartmental assessment (internal limiting membrane, ellipsoid zone [EZ], retinal pigment epithelium [RPE], and Bruch’s membrane). Two certified OCT readers independently completed manual segmentation and B-scan level validation of automated segmentation, with segmentation correction when needed (semi-automated). Certified reader metrics were compared to gold standard metrics using intraclass correlation coefficients (ICCs) to assess overall agreement. Across different diseases, several metrics generated from automated segmentations approached or matched human readers performance. Results: Absolute ICCs for retinal mean thickness measurements showed excellent agreement (range 0.980–0.999) across four cohorts. EZ-RPE thickness values and sub-RPE compartment ICCs demonstrated excellent agreement (ranges of 0.953–0.987 and 0.944–0.997, respectively) for full dataset, dry-AMD, and wet-AMD cohorts. Conclusions: Analyses demonstrated high reliability and consistency of segmentation of outer retinal compartmental features using a completely human/manual approach or a semi-automated approach to segmentation. These results support the critical role that measuring features, such as photoreceptor preservation through EZ integrity, in future clinical trials may optimize clinical care.

## 1. Introduction

Spectral-domain optical coherence tomography (SD-OCT) is a non-invasive imaging modality capable of acquiring high-resolution, three-dimensional, cross-sectional images of retinal tissue and is widely used in the management of retinal diseases. Accurate quantification of layer thicknesses with compartmental assessment and pathologic feature assessment (i.e., fluid volumes) is critical for evaluating disease severity and identifying potential biomarkers for disease progression and treatment response [1,2,3]. Historically, many of these advanced analyses were not feasible, such as measuring the ellipsoid zone (EZ) integrity due to the work burden and lack of consistency across readers. Progressive EZ attenuation is recognized as a surrogate for photoreceptor loss. Both EZ attenuation and photoreceptor loss precede and predict the progressive pathological changes associated with vision loss and age-related macular degeneration AMD [2]. AMD preferentially affects the macular (central) region of the retina and is characterized as early, intermediate, or late stages based on number, location, and size of drusen with hyper- or hypopigmentary changes and the presence or absence of geographic atrophy (GA) or macular neovascularization (MNV) [4]. Observed EZ changes are typically associated with the eventual loss of the retinal pigment endothelium (RPE) and the underlying choriocapillaris, leading to areas of GA [5]. Manual layer and pathologic feature segmentation by trained readers is both time-consuming and may be at risk to increased variability, limiting its utility and application within the field. Multiple automated segmentation algorithms have been developed to analyze OCT images across a variety of retinal diseases [6,7,8,9,10,11,12,13,14,15,16,17,18,19,20]. The capabilities of these various algorithms across disease spectrums can be highly variable and their ability to assess specific features of interest, such as EZ integrity, remains unclear. For current clinical trials, human validation and correction of any automated-generated segmentation is critical from a regulatory standpoint in the absence of an approved device for creating the segmentation of interest. Evaluation of image segmentation algorithms is often based on pixel-level classification, where the classification produced by the algorithm is compared to a ground-truth classification created by human readers and quantified using a wide variety of metrics [21]. In image segmentation in particular, spatial overlap indices, such as the Dice Similarity Coefficient (F1 score), are widely utilized to evaluate performance [22].

Regardless of the performance metrics chosen, a lack of established thresholds for defining acceptable performance makes it difficult to determine the feasibility of the performance of these algorithms in their use for fully automated image analysis. To address the challenge of defining acceptable performance, many studies compare the algorithm’s metrics to those from human readers, and a select number of studies have also compared clinical metrics in the form of layer thicknesses and fluid volumes from individual images obtained by the algorithm to those obtained by humans [23,24,25]. OCT segmentation is frequently utilized to obtain clinically relevant measures of disease burden (e.g., retinal volumes) and traditional performance metrics obtained from pixel classification do not directly assess how reliably an algorithm is able to reproduce these clinically relevant measures at the level of volumetric OCT scans when compared to human readers.

Traditionally, OCT segmentation and compartmental analysis have been primarily limited to overall retinal thickness measurements (e.g., internal limiting membrane [ILM] to retinal pigment epithelium [RPE]), which have only a limited correlation to visual function. Compartmental assessment, such as EZ integrity (i.e., EZ-RPE thickness), has shown significant association with functional measures, making these measurements viable targets and reliable clinical trial endpoints [3,26,27,28,29,30]. Although fully automated assessment has particular appeal in real-world utilization, clinical trial assessments are still grounded based in human certified-reader validation of segmentation. In fact, based on current regulatory requirements, purely automated assessments are generally not allowed, partly due to limited validation of these technologies. In addition, many current reading centers that use strictly human-based image interpretation segmentation greatly limit the specific targeted areas of pathology and features that can be characterized in clinical trials. Fluid features, for example, are often considered present/absent rather than quantified volumetrically. This loss of critical granularity of disease activity may impact understanding of clinical trial results and also limit generalizability to specific patients for treatment decision-making.

Emerging technologies utilizing machine learning are enhancing segmentation feasibility and scalability for multiple retinal features, including EZ integrity [30,31,32,33,34,35]. One key opportunity with this technology is to provide interpretable outputs that can be easily validated and corrected by expert certified readers. It is also important to understand differences that may occur in segmentation based on the specific disease that is being evaluated. In addition, based on the association between EZ loss and GA progression, imaging photoreceptor degeneration may serve as a prognostic biomarker and an attractive clinical end point in future studies evaluating novel therapies for dry AMD. This may also contribute to addressing the progressive decline in visual function experienced by patients with AMD. In this analysis, we compared clinical measurements obtained from both completely manual segmentation approaches and a semi-automated approach to segmentation in eyes with dry AMD, wet AMD, and diabetic macular edema (DME).

## 2. Materials and Methods

### 2.1. Image Selection

This was an institutional review board (IRB)-exempt, as determined by the Cleveland Clinic IRB, image analysis of 210 independent macular cube scans utilized for this segmentation quality assessment. The dataset included 60 dry AMD, 60 wet AMD, 60 DME, and 30 normal scans without pathology, for a total of 23,625 B-scans with 7875 B-scans from the central 2 mm. Macular cube scans obtained from Zeiss (Oberkochen, Germany) Cirrus (scan pattern: 512 × 128; scan area: 6 × 6 mm) and Heidelberg (Heidelberg, Germany) Spectralis (scan patterns: 1024 × 97 and 512 × 97; scan area: 6 × 6 mm) SD-OCT devices were included in each of the four cohorts in a 1:1 ratio. All scans were screened for image quality. In the dry AMD, wet AMD, and DME cohorts, scans were selected to ensure a wide spectrum of disease burden was represented. All dry AMD scans featured drusen, geographic atrophy (GA), or both; all DME scans contained intraretinal fluid (IRF); all wet AMD scans featured IRF, subretinal fluid (SRF), subretinal pigment epithelium fluid (subRPE fluid), and/or subretinal material (SRM) alone or, in many cases, in combination.

### 2.2. Manual Image Segmentation

All certified readers involved in this study underwent the standard intense training program for the Tony and Leona Campane Center for Excellence in Image-Guided Surgery and Advanced Imaging Research at the Cleveland Clinic. This consisted of approximately 200 h of training on the segmentation approach to various retinal layers and diseases, including the diseases involved in this analysis. As per protocol for our reading center, all room lighting was standardized, no remote reading was performed, and all computer monitors were standardized.

A manual segmentation protocol where readers segmented each scan without any baseline segmentation was completed using the OCTViewer (Cleveland Clinic, Cleveland, OH, USA) segmentation platform with the manual editing function [3,18,29,36]. The manual segmentation was performed to evaluate whether the algorithm is able to produce metrics comparable to humans and provide an unbiased reference point for the results of the semi-automated segmentation. Two certified readers performed manual segmentation for all retinal layers of interest (ILM, EZ, RPE, and BM). The same two certified readers performed the analysis for all scans. These layers were segmented through the center of the layer of interest. In eyes without RPE elevation, BM was segmented at the bottom of the RPE. In areas without EZ visible, the EZ was dropped to the RPE band (for an effective EZ-RPE thickness of 0 microns), or if subretinal fluid or subretinal material was present, it was dropped to the interface of the retina and the SRF/SRM if it was not visible within the retinal tissue. Two additional senior certified readers worked collaboratively to create a gold standard segmentation set. For each gold standard scan, the segmentation was divided between the two gold standard readers who each completed a portion of the segmentation and then reviewed and validated the other reader’s segmentation (consensus segmentation). As an example, in the DME dataset, the first gold standard reader completed the segmentation of the ILM, RPE, and BM for scans #1–30 and the EZ and fluid for scans #31–60, while the other reader segmented the EZ and fluid for scans #1–30 and the ILM, RPE, and Bruch’s membrane (BM) for scan #31–60. This consensus approach to the development of the gold standard was utilized to minimize the impact of any biases from a single reader.

Given the extensive labor burden for comprehensive pathology/layer segmentation, manual segmentation was restricted to the central macular subfield (central 2 mm) of each scan. This area was selected given the high incidence of foveal pathology and the often more limited peripheral pathology present. Manual segmentation of the entire dataset was completed prior to starting the semi-automated segmentation so that the readers would not be biased by viewing the deep-learning platform’s baseline segmentation.

### 2.3. Semi-Automated Segmentation—Fully Automated Initial Segmentation with Sequential Human Line-by-Line Validation

All macular cube scans were imported into a retinal layer segmentation platform, OCTViewer, which provided initial fully automated, machine learning-enhanced segmentation of the ILM, EZ, RPE, and Bruch’s membrane (BM) [3,18,29,36].

This platform utilizes multiple convolutional neural network models with integrated logic based on segmentation findings: (1) a fluid model; (2) a low-magnification layer model; and (3) a high-magnification layer model, each with a similar U-net style architecture [37]. In addition to the fluid segmentation model, a higher order classifier is applied to identify the type of fluid that is present following the initial segmentation. The low-magnification and fluid models were trained using 10 × 10 convolutional kernels to allow for more contextual information than the standard 3 × 3 convolutional models, while the high-magnification models were trained using 12 × 12 convolutional kernels [38]. The high-magnification layer models operated at a higher resolution, using cropped areas around the bounds identified by the low-magnification layer models to enable more precise segmentation. A binary cross entropy training loss function was utilized along with an Adam optimizer and a loss rate of 1 × 10^−4^ [36].

Similarly to the manual segmentation, two certified readers independently reviewed the segmentation outputs and made manual corrections when necessary (i.e., segmentation errors). Two senior certified readers worked collaboratively to create a gold standard semi-automated segmentation set as described in the manual segmentation section. The semi-automated segmentation was performed to assess whether or not the edits that readers made to each scan resulted in meaningful changes in the layer and fluid metrics.

### 2.4. Segmentation Metrics

For comparative assessment between the manual and semi-automated analyses, mean layer thickness along with fluid volume metrics for the central macular region (central 2 mm diameter foveal-centered zone) were exported for each scan for both the manual and semi-automated segmentations. The full list of metrics selected to evaluate platform performance is presented in Table 1.

### 2.5. Statistical Analysis

A full cohort analysis utilizing all 210 scans was conducted for the layer thickness metrics. Separate disease-specific cohort analyses were conducted to evaluate layer segmentation performance in the different diseases and to assess performance for fluid metrics relevant to specific diseases. This internal quality control cohort of scan samples was assembled from images available within the Tony and Leona Campane Center of Excellence of Image-Guided Surgery and Advanced Imaging Research. The underlying diagnosis had been previously validated by a retinal disease expert. The reader 1 (R1) and reader 2 (R2) metrics were compared to the gold standard metrics in order to evaluate the conditions under which the algorithm was able to reproduce metrics similar to human readers. To summarize the agreement between each pair, intra-class correlation coefficients (ICCs) were calculated using the SPSS Reliability Analysis tool (IBM, Armonk, NY, USA; Version 28.0, 2021). Absolute agreement and consistency ICCs were calculated using the two-way random effects model with single measures. Values less than 0.5 are indicative of poor reliability; values between 0.5 and 0.75 indicate moderate reliability; values between 0.75 and 0.9 indicate good reliability; and values greater than 0.90 indicate excellent reliability [39]. Both the absolute agreement and consistency forms of the ICC were calculated as some of the data distributions revealed the presence of fixed biases [40]. Sample sizes were chosen based on preliminary data to constrict the width of the confidence intervals around the ICCs to ±0.1 with an alpha level of 0.05 [41].

## 3. Results

### 3.1. Full Cohort

Absolute agreement and consistency ICCs for the full cohort are shown in Table 2. For ILM-RPE thickness, manual certified reader agreement demonstrated excellent agreement/consistency with ICCs from 0.998 to 0.999. Similar performance was seen in the semi-automated approach. For EZ integrity measures based on EZ-RPE thickness, excellent agreement/consistency was also seen with ICCs from 0.965 to 0.986 across both manual and semi-automated approaches. For the subRPE compartment (i.e., RPE-BM thickness measures), the ICCs also exhibited excellent agreement with a range from 0.983 to 0.997.

### 3.2. Dry AMD Cohort

For the dry AMD cohort, absolute agreement and consistency ICCs are shown in Table 3. For ILM-RPE thickness, manual certified reader agreement demonstrated excellent agreement/consistency with ICCs ranging from 0.996 to 0.995. Similar performance was seen in the semi-automated approach. For EZ integrity measures in dry AMD, excellent agreement/consistency was also seen in EZ-RPE thickness with ICCs ranging from 0.967 to 0.9868 across both manual and semi-automated approaches. For the subRPE compartment in dry AMD, including both drusen and geographic atrophy (i.e., RPE-BM thickness measures), the ICCs also exhibited excellent agreement with a range from 0.944 to 0.950.

### 3.3. DME Cohort

For the DME cohort, absolute agreement and consistency ICCs are shown in Table 4. For ILM-RPE thickness, manual certified reader agreement demonstrated excellent agreement/consistency with ICCs ranging from 0.998 to 0.999. Similar performance was seen in the semi-automated approach. For EZ integrity measures in DME, manual agreement/consistency ranged from 0.842 to 0.905. Semi-automated assessment demonstrated greater agreement/consistency with ICCs ranging from 0.955 to 0.969. The RPE-BM metrics for manual reader comparison for Reader 2 demonstrated poor agreement based on ICCs ranging from 0.107 to 0.108. Reader 1 demonstrated better agreement with a range of 0.521–0.726. This may be related to the challenges of consistent RPE/BM segmentation in eyes that have minimal anatomic disturbance. The semi-automated approach demonstrated dramatically better agreement and consistency with ICCs ranging from 0.907 to 0.925.

### 3.4. Wet AMD Cohort

In wet AMD, absolute agreement and consistency ICCs are shown in Table 5. For overall retinal thickness measures (i.e., ILM-RPE thickness), manual certified reader agreement demonstrated excellent agreement/consistency with ICCs ranging from 0.995 to 0.997. The semi-automated approach also exhibited excellent agreement with ICCs ranging from 0.980 to 0.985. For EZ integrity measures in wet AMD, EZ-RPE thickness values demonstrated excellent agreement/consistency with ICCs ranging from 0.953 to 0.987 across both manual and semi-automated approaches. For the subRPE compartment in wet AMD (i.e., RPE-BM thickness measures), the ICCs also exhibited excellent agreement with a range from 0.944 to 0.950.

### 3.5. Gold Standard Manual vs. Semi-Automated Segmentation

Absolute agreement ICCs for the full cohort layer thickness metrics comparing the gold standard manual values to the gold standard semi-automated values showed excellent agreement ranging from 0.965 to 0.994, demonstrating stability across approaches.

## 4. Discussion

In this analysis, we evaluated the feasibility, agreement, and consistency of completely manual segmentation and semi-automated segmentation of outer retinal compartmental measurements, including EZ integrity (i.e., EZ-RPE thickness) and the subRPE compartment (i.e., RPE-BM) across multiple retinal conditions. There was comparable consistency of a fully automated, deep learning-augmented segmentation platform, as validated by human readers (semi-automated), to expert human readers. We utilized clinically relevant measures of disease burden in the form of layer thicknesses and pathology-specific fluid volumes and assessed agreement using ICCs rather than the traditional pixel-classification-based performance metrics utilized in many image segmentation studies [20]. An alternative to ICCs, would be to also evaluate the data using a Bland–Altman plot provides a quantitative estimate of how closely the values from two measurements lie and provides a graphical representation of the data.

Our analysis also included both an unbiased manual segmentation protocol as well as a semi-automated segmentation protocol, the current gold standard, to assess measurement performance. Furthermore, we evaluated the segmentation of multiple retinal layers across multiple retinal diseases with each disease cohort, including scans with mild-to-severe pathology from multiple OCT device vendors as would be encountered in large-scale clinical trials. Previous studies have assessed the performance of automated algorithms in segmenting retinal layers in single diseases on a single device [21], but to our knowledge, no prior studies have assessed both layer and fluid segmentation across multiple diseases, with each disease cohort containing images from multiple OCT vendors and with both manual and semi-automated comparative assessments.

Whether using traditional image processing for initial segmentation or machine-learning enhanced segmentation, semi-automated segmentation with expert human validation/correction is the current gold standard in clinical trials. Since the readers were not masked to the purposes of the study, the level of agreement from the semi-automated analysis could be called into question by arguing that the readers may have been biased to make minimal edits to the automated segmentation. The manual segmentation analysis was included to provide an unbiased performance assessment and evaluate the feasibility of segmentation consistency and agreement for highly challenging pathologic/OCT features, such as EZ integrity, in diseases, such as dry AMD. It also highlighted some interesting findings related to outliers for performance on manual segmentation, particularly in eyes with minimal pathology. Specifically, in eyes with minimal RPE-BM pathology (i.e., DME), manual segmentation demonstrated significantly worse ICC performance, particularly compared to the semi-automated approach. One contributing factor to the lower ICC is the lack of variability in the thickness values in the DME sample, which is due to a lack of pathology in the RPE-BM complex. On segmentation review, it also appeared that one of the readers consistently manually placed the segmentation lines closer together in eyes without pathology.

Our study had some important limitations. Images with quality defects were excluded from the analysis, limiting the generalizability of our findings across all scan qualities. Furthermore, while each pathology was represented by scans ranging in severity, with 60 scans in each disease cohort, there may have been features of each disease not captured within our datasets. Additional ongoing work is being completed to further evaluate next-generation machine-learning-enhanced segmentation platforms that integrate pathology-based decision-making and to evaluate a more extensive metrics portfolio.

This analysis provides promising data supporting the feasibility of high agreement/consistency for metrics related to outer retinal features, including EZ integrity (i.e., EZ-RPE thickness) and the subRPE compartment (i.e., RPE-BM thickness), comprising both drusen and geographic atrophy, using either a purely manual approach or semi-automated assessment consisting of initial automated segmentation with a machine-learning enhanced multi-layer segmentation and subsequent human overreads. The value of the underlying high-intensity training of the certified readers involved in this process, which included over 200 h of training focused specifically on high-pathology segmentation of complex retinal features, such as EZ integrity, should also be considered. The scalability and reliability of these measurements are now enabling these parameters to be used as endpoints in large-scale clinical trials, which may fulfill the unmet need to address the decline in visual function experienced by patients with AMD.

## Figures and Tables

**Table 1:** Retinal compartment and pathologic feature definitions.

Metrics	Description
Retinal (ILM-RPE) Thickness	Mean thickness between the ILM and RPE
EZ-RPE Thickness	Mean thickness between the EZ and RPE
RPE-BM Thickness	Mean thickness between the RPE and BM

Abbreviations: BM = Bruch’s membrane; EZ = ellipsoid zone; ILM = inner limiting membrane; RPE = retinal pigment epithelium.

**Table 2:** Full cohort ICCs.

	Manual		Semi-Automated	
	Reader 1	Reader 2	Reader 1	Reader 2
ILM-RPE				
Agreement	0.998	0.999	0.996	0.995
Consistency	0.998	0.999	0.996	0.995
EZ-RPE				
Agreement	0.984	0.968	0.979	0.965
Consistency	0.986	0.970	0.979	0.965
RPE-BM				
Agreement	0.997	0.989	0.992	0.983
Consistency	0.997	0.990	0.992	0.983

Agreement: Two-way random effects, single measures, absolute agreement ICC. Consistency: Two-way random effects, single measures, consistency ICC. ILM-RPE Thickness: Mean thickness between the ILM-RPE for the central 2 mm fovea-centered subfield. EZ-RPE: Mean thickness between the EZ and RPE for the central 2 mm fovea-centered subfield. RPE-BM: Mean thickness between the RPE and Bruchs membrane for the central 2 mm fovea-centered subfield. Abbreviations: BM = Bruch’s membrane; EZ = ellipsoid zone; ICC = intraclass correlation coefficients; ILM = inner limiting membrane; RPE = retinal pigment epithelium.

**Table 3:** Dry AMD ICCs.

	Manual		Semi-Automated	
	Reader 1	Reader 2	Reader 1	Reader 2
Dry AMD Layer Metric ICCs				
ILM-RPE				
Agreement	0.996	0.995	0.998	0.999
Consistency	0.996	0.995	0.998	0.999
EZ-RPE				
Agreement	0.977	0.967	0.979	0.988
Consistency	0.977	0.967	0.979	0.988
RPE-BM				
Agreement	0.987	0.944	0.980	0.991
Consistency	0.987	0.950	0.985	0.991

Agreement: Two-way random effects, single measures, absolute agreement ICC. Consistency: Two-way random effects, single measures, consistency ICC. Abbreviations: AMD = age-related macular degeneration; BM = Bruch’s membrane; EZ = ellipsoid zone; ICC = intraclass correlation coefficients; ILM = inner limiting membrane; RPE = retinal pigment epithelium.

**Table 4:** DME layer ICCs.

	Manual		Semi-Automated	
	Reader 1	Reader 2	Reader 1	Reader 2
DME Layer Metric ICCs				
ILM-RPE				
Agreement	0.998	0.999	1.000	1.000
Consistency	0.998	0.999	1.000	1.000
EZ-RPE				
Agreement	0.842	0.876	0.968	0.955
Consistency	0.865	0.905	0.969	0.957
RPE-BM				
Agreement	0.521	0.107	0.907	0.924
Consistency	0.726	0.108	0.909	0.925

Agreement: Two-way random effects, single measures, absolute agreement ICC. Consistency: Two-way random effects, single measures, consistency ICC. ILM-RPE Thickness: Mean thickness between the ILM-RPE for the central 2 mm fovea-centered subfield. EZ-RPE: Mean thickness between the EZ and RPE for the central 2 mm fovea-centered subfield. RPE-BM: Mean thickness between the RPE and Bruchs membrane for the central 2 mm fovea-centered subfield. Abbreviations: BM = Bruch’s membrane; DME = diabetic macular edema; EZ = ellipsoid zone; ICC = intraclass correlation coefficients; ILM = inner limiting membrane; RPE = retinal pigment epithelium.

**Table 5:** Wet AMD Layer ICCs.

	Manual		Semi-Automated	
	Reader 1	Reader 2	Reader 1	Reader 2
Wet AMD Layer Metric ICCs				
ILM-RPE				
Agreement	0.996	0.995	0.985	0.980
Consistency	0.997	0.997	0.985	0.980
EZ-RPE				
Agreement	0.986	0.963	0.972	0.953
Consistency	0.987	0.968	0.972	0.953
RPE-BM				
Agreement	0.997	0.988	0.990	0.978
Consistency	0.997	0.991	0.990	0.978

Agreement: Two-way random effects, single measures, absolute agreement ICC. Consistency: Two-way random effects, single measures, consistency ICC. ILM-RPE Thickness: Mean thickness between the ILM-RPE for the central 2 mm fovea-centered subfield. EZ-RPE: Mean thickness between the EZ and RPE for the central 2 mm fovea-centered subfield. RPE-BM: Mean thickness between the RPE and Bruchs membrane for the central 2 mm fovea-centered subfield. Abbreviations: BM = Bruch’s membrane; EZ = ellipsoid zone; ICC = intraclass correlation coefficients ILM = inner limiting membrane; RPE = retinal pigment epithelium.

## Data Availability

The raw data supporting the conclusions of this articles will be made available by the authors on request.
